# Overexpression of glutathione synthetase gene improving redox homeostasis and chicken infectious bursal disease virus propagation in chicken embryo fibroblast DF-1

**DOI:** 10.1186/s40643-023-00665-0

**Published:** 2023-09-09

**Authors:** Jia Lin, Rui Min, Xiaoping Yi, Yingping Zhuang

**Affiliations:** 1 State Key Laboratory of Bioreactor Engineering, East China University of Science and Technology (ECUST), 130 Meilong Rd., Shanghai, 200237 People’s Republic of China; 2 Collaborative Innovation Center for Rehabilitation Technology, The Academy of Rehabilitation Industry, Fujian University of Traditional Chinese Medicine, Fuzhou, People’s Republic of China

**Keywords:** Chicken infectious bursal virus, Chicken embryonic fibroblasts DF-1, Glutathione, Glutathione synthetase

## Abstract

Infectious bursal disease (IBD) of chickens is an acute, high-contact, lytic infectious disease caused by infectious bursal disease virus (IBDV). The attenuated inactivated vaccine produced by DF-1 cells is an effective control method, but the epidemic protection demands from the world poultry industry remain unfulfilled. To improve the IBDV vaccine production capacity and reduce the economic losses caused by IBDV in chicken, cellular metabolic engineering is performed on host cells. In this study, when analyzing the metabolomic after IBDV infection of DF-1 cells and the exogenous addition of reduced glutathione (GSH), we found that glutathione metabolism had an important role in the propagation of IBDV in DF-1 cells, and the glutathione synthetase gene (*gss*) could be a limiting regulator in glutathione metabolism. Therefore, three stable recombinant cell lines GSS-L, GSS-M, and GSS-H (*gss* gene overexpression with low, medium, and high mRNA levels) were screened. We found that the recombinant GSS-M cell line had the optimal regulatory effect with a 7.19 ± 0.93-fold increase in IBDV titer. We performed oxidative stress and redox status analysis on different recombinant cell lines, and found that the overexpression of *gss* gene significantly enhanced the ability of host cells to resist oxidative stress caused by IBDV infection. This study established a high-efficiency DF-1 cells system for IBDV vaccine production by regulating glutathione metabolism, and underscored the importance of moderate gene expression regulation on the virus reproduction providing a way for rational and precise cell engineering.

**Graphical Abstract:**

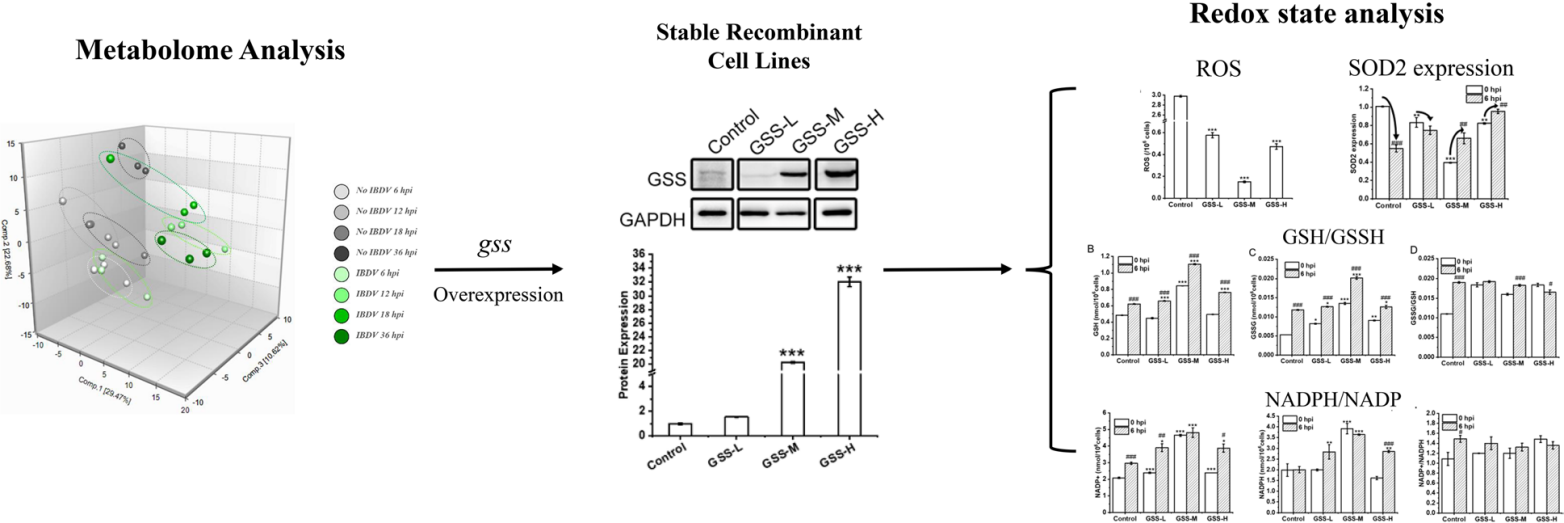

**Supplementary Information:**

The online version contains supplementary material available at 10.1186/s40643-023-00665-0.

## Introduction

Infectious bursal disease in chickens has been found in flocks for more than 50 years now and is an acute, high-contact, lytic infectious disease caused by the infectious bursal virus IBDV, which is a small-molecule, envelope-free virus belonging to the genus *Avibirnavirus* of the *Birnaviridase* family (Leong 2000). IBDV also exacerbates other viral infections and causes huge economic losses to the world poultry industry (Müller et al. 2003). An attenuated inactivated vaccine is an effective control method for this disease, and DF-1 cell is useful for IBDV vaccine production (Choi et al. 2020). We found that the process of IBDV propagation in DF-1 cells would affect the metabolism of the host cells since infection (Lin et al. 2020). There were mainly eight cell metabolic pathways that changed (Rodrigues et al. 2013): amino acid catabolism, carbohydrate catabolism and the integration of energy metabolism, nucleotide metabolism, pentose phosphate pathway, polyamine biosynthesis, lipid metabolism, and glutathione metabolism. Here, we found that the changes in glutathione metabolism in DF-1 cells caused by IBDV invasion play an important role in virus reproduction.

The altered glutathione metabolism of host cells is associated with intense oxidative stress during virus infection, mainly manifested as the imbalance between oxidative and antioxidant effects in vivo. Oxidants and hyperoxia radicals, especially the reactive oxygen species (ROS), are the main agents acting in oxidative stress and have an important role in the pathogenesis of various infectious diseases. There are two types of antioxidant systems in cells. One is the enzymatic antioxidant system, with superoxide dismutase (SOD) as the main marker (He et al. 2016), and the other is the non-enzymatic antioxidant system, with glutathione as the main marker (Espinosa-Diez et al. 2015). Glutathione is available in both reduced (GSH) and oxidized (GSSG) forms. GSSG/GSH ratio can reflect the redox state since GSH is transferred into GSSG when cells undergo oxidative stress. A controlled intracellular glutathione redox cycle is a guarantee for maintaining a favorable intracellular redox state (Schafer and Buettner 2001). NADP+/NADPH is a cofactor pair that provides active sources of protons and electrons and is closely linked with GSSG/GSH. GSH, together with NADPH and related enzymes, form a complex antioxidant network that is involved in maintaining the redox state of the organism (Ouyang et al. 2018; Ye et al. 2015; Lu and Holmgren 2014).

Glutathione, the main acting substrate of cellular resistance to oxidative stress, involves several enzymes in its metabolism (Tsugawa et al. 2019), among which glutathione synthase (GSS) catalyzes the synthesis of reduced glutathione from γ-glutamylcysteine and glycine in an ATP-dependent manner (Njalsson et al. 2001), and the activity of GSS is linearly correlated with the intracellular content of GSH (Dickinson and Forman 2002). Moore et al*.* recombinantly expressed GSS and γ-glutamylcysteine synthetase (γ-GCS) proteins in *E. coli*, which significantly promoted glutathione concentrations, and hypothesized that GSH synthesis in mammals facilitates cellular resistance to toxic substances (Moore et al. 1989). Volohonsky et al*.* abstracted GSS and γ-GCS proteins in organs or cells such as murine liver and kidney and found that γ-GCS was feedback inhibited by GSH, while GSS was non-restricted enzyme and not feedback inhibited by GSH (Volohonsky et al. 2002). GSS can modulate the GSH redox system by increasing GSH synthesis to resist oxidative stress in harsh environments (ZhuY et al. 1999; Li et al. 2006). Despite the low amino acid sequence homology of GSS among different species, they have important roles in cell growth (Jez 2019). Therefore, overexpression of GSS has the potential to regulate intracellular GSH concentration to promote cell growth and viral productivity.

In this study, we analyzed the metabolomic of DF-1 cells after IBDV infection, and explored the importance of glutathione pathway by the exogenous addition of GSH. Then, we overexpressed the *gss* gene, screened recombinant DF-1 cell lines with different gene expression levels, and further evaluated the virus reproduction capacity and redox status of recombinant cell lines after IBDV infection.

## Materials and methods

### DF-1 cells and IBDV culture

Routine cell culture of adherent DF-1 cells was performed in Nunc EasyFlask 25 cm^2^ (Thermo Scientific) with 5 ml DMEM/F12 (1:1) with 5% fetal bovine serum (Biological Industries) in a humidified incubator at 37 ℃ with 5% CO_2_. Cell number and viability were determined using Countstar (ALIT Life Science), an automated trypan blue cell counter. The specific growth rate (h^−1^) was calculated as the following equation:

$$\mu =\frac{Ln{X}_{2}-Ln{X}_{1}}{{t}_{2}-{t}_{1}},$$

where *t*_1_ and *t*_2_ were the culture time (h), *X*_1_ and *X*_2_ were the corresponding cell concentration (cells/ml).

IBDV multiplied in DF-1 cell culture provided by our lab was used throughout this study. IBDV infected DF-1 in the flask when cells reached a confluence of 90% (about 36 h of DF-1 growth) and harvested when 80% of cells were observed lesions (about 36 hpi after IBDV infection), and then determined virulence using TCID_50_ as stated in the previous report (Lin et al. 2020). The relative titer was calculated as the following equation:

$$\text{Relative Titer}= {10}^{\left[{\text{log}}_{10}\left({\text{TCID}}_{\text{50,sample}}\right)-{\text{log}}_{10}\left({{\text{TCID}}}_{\text{50,control}}\right)\right]},$$

where TCID_50, control_ is the average virus titer at 36 hpi in control group, and TCID_50, sample_ is the recombinant group.

### Metabolomics analysis

The metabolome experiments have been shown in the previous reports (Lin et al. 2020). In short, the metabolites in DF-1 cells infected by IBDV at 6,12,18, 36 hpi were determined by Metabolon, Inc. (Durham, NC) using standard protocols with triple duplication in each group (Lawton et al. 2008). All identified metabolite relative abundance matrices were uploaded on MetaboAnalysis (http://www.metaboanalyst.ca) for topological analysis (Chong 2018; Xia and Wishart 2016).

### Construction of gss overexpression DF-1 cell line

EcoR I and Xba I were digestion sites at the 5’ and 3’ ends of the chicken *gss* gene (NCBI number XM_425692.5) in the pCI-neo vector (Additional file 1: Fig. S1A). DF-1 cells were seeded in 24-well plates at a concentration of 3 × 10^5^ cells/well 16 h prior to being 70–90% confluent at transfection. Plasmid DNA (1 μg) expressing the *gss* gene was transfected into DF-1 cells mediated by Lipofectamine 3000 (Invitrogen) as the manual. The fresh medium with 800 μg/ml G418 (Sigma) was replaced every 48 h until the cell growth was stable and the cells without transfection were dead. The cell clones were screened in 96-well plates. DF-1 cells expressing lacZ were set as control.

### RNA isolation and quantitation RT-PCR

Total RNA was isolated from DF-1 cells using the TRIzol extraction method as described previously. Purified RNAs were eluted using 20 μl RNase-free water and stored in a − 80 ℃ freezer. The quality and quantity of RNA were evaluated using a spectrophotometer (NanoDrop 2000, Thermo Scientific). For cDNA synthesis, RNA was reverse-transcribed using the First Strand cDNA Synthesis kit (Thermo Scientific) according to the manufacturer’s instructions with Oligo(dT)_18_ primers after RNase-free DNase treatment. For gene expression analysis, the sequences of forward and reverse primers used to amply chicken *gss*, *gsr*, *ggt*, *sod2,* and housekeeping gene β-actin were designed by NCBI blast (Table 1). The cDNA samples were amplified in triplicate by real-time qPCR using TB Green Premix Ex Taq II (Takara) and the CFX96 Touch Real-time PCR Detection System (Bio-Rad Laborites, Inc.). Gene expression levels were estimated based on PCR efficiency and threshold cycle (Ct) deviation of an unknown sample vs. a control.

**Table 1:** The primers used in this study

Primers	Sequences	Production length (bp)
qGSS-F	GAGCTTTGGGACAGGAACAT	138
qGSS-R	CACGTAGCCCTCTCTGTAGT	
qGSR-F	TTCATCCACGACCATCCTGA	147
qGSR-R	GATGATGTCAATGTGAGCCTTG	
qGGT-F	CCATTGCTGGTTTGATCTG	188
qGGT-R	TTTGGGACCGATGTGTAAA	
qSOD2-F	AAGGAGCAGGGACGTCTACA	97
qSOD2-R	CCCATACATCGATTCCCAGCA	
GSS-F (EcoR I)	ATTGAATTCTTAGCTATTGTCCAATCGCCG	1591
GSS-R (Xba I)	GCTCTAGAGCCCAACAAATGCAAAACCATTG	

### Western blot

The cell protein extracts (10 μg) from control DF-1 cells and *gss* overexpressed cells by RIPA Lysis Buffers (Beyotime) were prepared by detecting their total protein concentration using Pierce^™^ BCA Protein Assay Kit (Thermo Scientific). Samples were subsequently subjected to SDS/PAGE one 7.5% (*w*/*v*) polyacrylamide gels. Proteins were transferred onto a nitrocellulose membrane. After blocking in QuickBlock^™^ Blocking Buffer for Western Blot (Beyotime), membranes were then probed with anti-GSS antibodies (ProteinTech; diluted to 1:1000) and detected with HRP-labeled goat anti-rabbit IgG (Abcam; diluted to 1:2000) after washing. GAPDH was used as a control.

### Redox-state analysis

 DF-1 cells were lysed by freeze–thaw method, and reduced/oxidized glutathione (GSH/GSSG) measurement was performed using a GSH and GSSG Assay Kit (Beyotime) according to the manufacturer’s protocol. NADP+ and NADPH levels of cells were detected with Enzychrom^™^ NADP+/NADPH Assay Kit (BioAssay Systems) according to the manufacturer’s protocol. Intracellular ROS level measurements were performed according to the Reactive oxygen species Assay Kit’s manufacturer’s protocol (Nanjing Jiancheng Bioengineering Institute).

### Statistical analysis

All experiments were repeated at least three times. The statistical significance of variables was evaluated by applying the analysis of variance (ANOVA) using Student’s *t* test. A *p*-value less than 0.05 was considered statistically significant and was indicated by an asterisk in the figures. Data were reported as mean ± standard deviation.

## Results

### Effect of glutathione metabolic pathway on IBDV replication based on metabolomics analysis

**Fig. 1:**
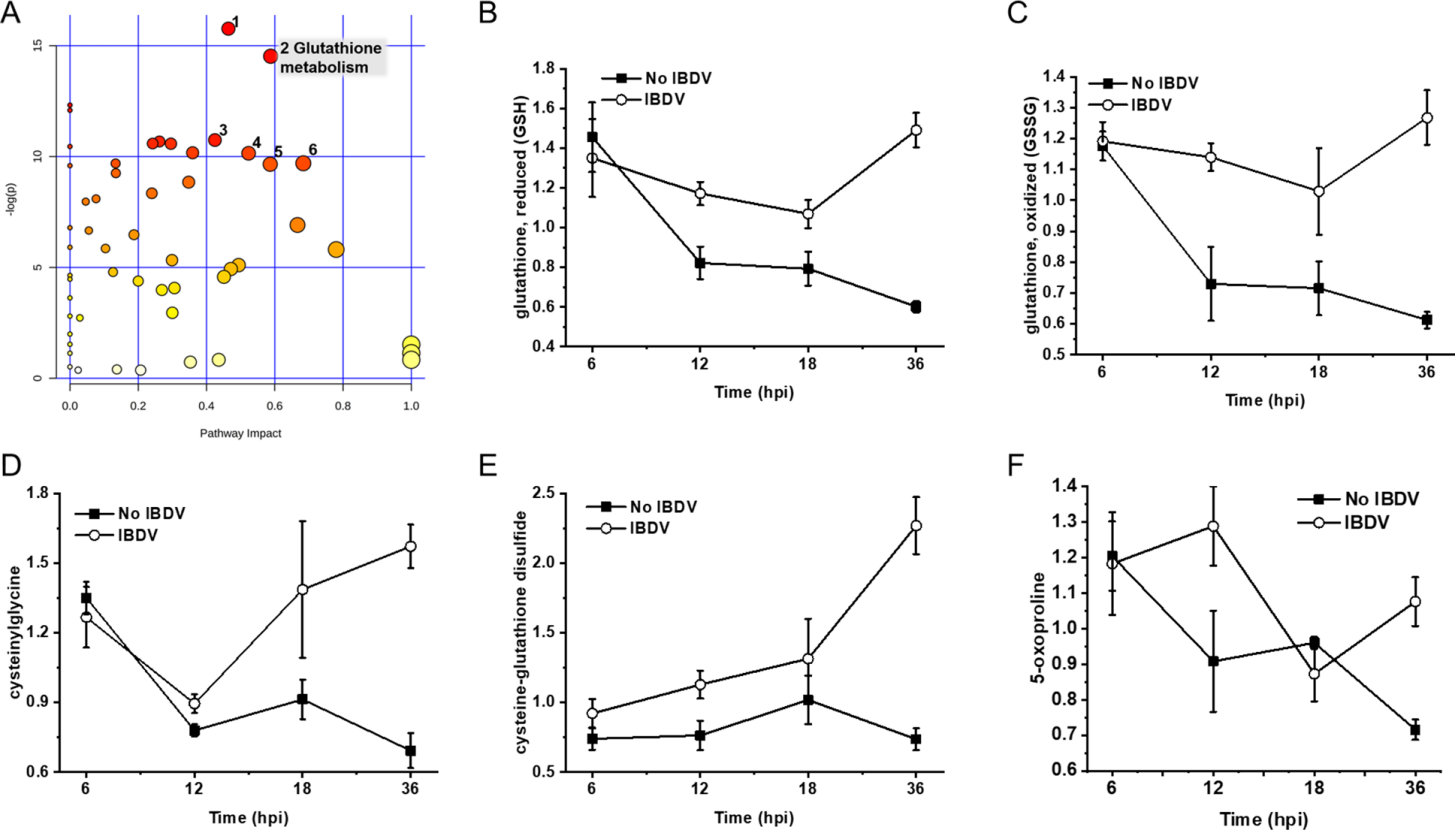
The relative intensity of metabolites in glutathione metabolism in DF-1 cells with IBDV incubation. **A** Topological analysis of the metabolic profiles in DF-1 cells induced by IBDV. **B**–**F** Show a significant increase in the intracellular intensities of GSH, GSSG, cysteinylglycine, cysteine-glutathione disulfide, and 5-oxoproline in DF-1 cells infected with IBDV (IBDV, open circle) for 12, 18 and 36 h compared with uninfected controls (no IBDV, solid square). Raw area counts from three independent experiments performed in triplicate (*N* = 3) were normalized to protein levels. Error bars show ± s.d. of the mean

In the metabolomic study after IBDV infection of DF-1 cells, intracellular metabolite intensities of DF-1 cells were examined at 0 hpi, 6 hpi, 12 hpi, 18 hpi, and 36 hpi after IBDV inoculation, with three parallels set at each time point, including the processes of IBDV infestation, replication, assembly, and secretion(Lin et al. 2020). By metabolic pathway topology analysis on the MetaboAnalyst, there were significant changes in the glutathione metabolic pathway during viral multiplication (Fig. 1A). The intracellular metabolites intensities associated with the glutathione metabolic pathway in DF-1 cells with IBDV were generally higher than the intracellular situation in DF-1 cells without IBDV inoculation (Fig. 1B–F). It is hypothesized that the upregulation of the glutathione metabolic pathway may facilitate the intracellular propagation of IBDV in DF-1 cells.

### Effect of exogenous addition of GSH on IBDV multiplication in DF-1 cells

**Fig. 2:**
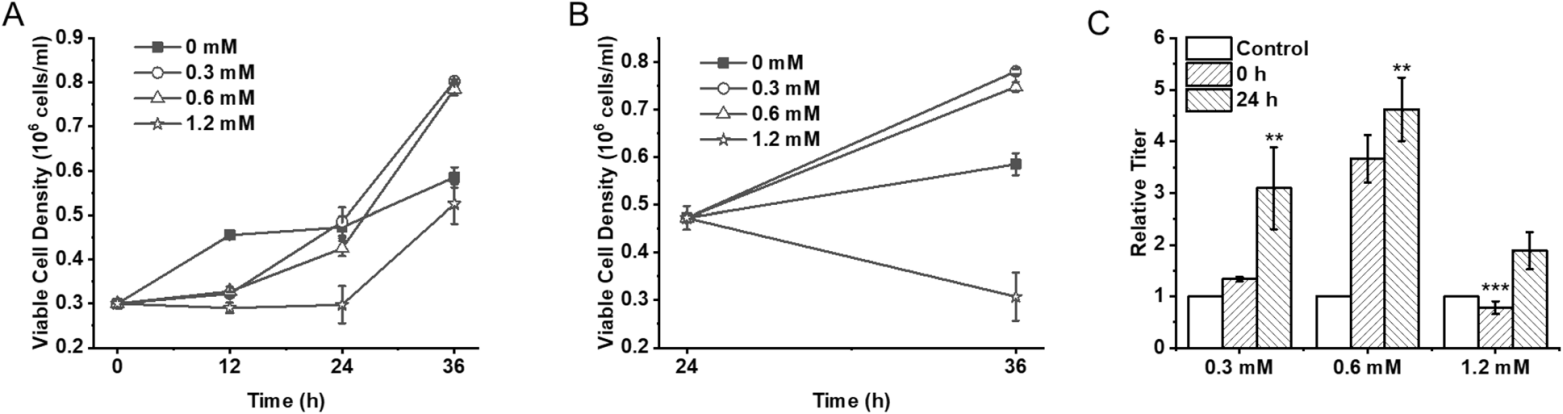
Effects of GSH in DF-1 cells and IBDV propagation. **A** DF-1 cells growth curve from 0 to 36 h with 0, 0.3, 0.6, 1.2 mM GSH addition at 0 h. **B** DF-1 cells growth curve from 24 to 36 h with 0, 0.3, 0.6, 1.2 mM GSH addition at 24 h. **C** The relative IBDV titers from DF-1 cells were treated with different GSH concentrations at 0 h and 24 h. The groups without GSH supplementation were set as the control. *N* = 3 biological replicates and error bars represent s.d. Asterisks “*” presented the differences between the control group (without GSH in medium) and the experimental groups (with 0.3, 0.6, 1.2 mM GSH in media). *p < 0.05, **p < 0.01, and ***p < 0.001 as determined by two-tailed *t* test

To investigate the effects of glutathione, 0 mM, 0.3 mM, 0.6 mM, and 1.2 mM of GSH were added at 0 h and 24 h during DF-1 cell growth, and then DF-1 cell growth and IBDV multiplication were examined (Fig. 2). The results showed that GSH could inhibit the early growth of DF-1 cells. Specifically, the exogenous addition of 0.3 mM and 0.6 mM GSH had a significant promotion effect on the growth of DF-1 cells when the cells grew to 24 h and started to enter the logarithmic growth phase (Fig. 2A, B). In addition, the addition of 0.6 mM GSH significantly promoted the acquisition of IBDV, 0.3 mM GSH had the second highest effect, while 1.2 mM GSH inhibited the propagation of IBDV. In addition, the growth state of the cells at the time of GSH addition also significantly affected IBDV propagation, and the IBDV titers obtained by adding GSH at the early growth stage of DF-1 cells were significantly lower than those obtained by adding GSH after the DF-1 cells entered the logarithmic growth stage (Fig. 2C). It indicates that the influence of exogenous GSH addition on IBDV propagation in DF-1 cells is not only concentration-dependent, but also time-dependent.

### The limitation of glutathione metabolism in DF-1 cells infected by IBDV

**Fig. 3:**
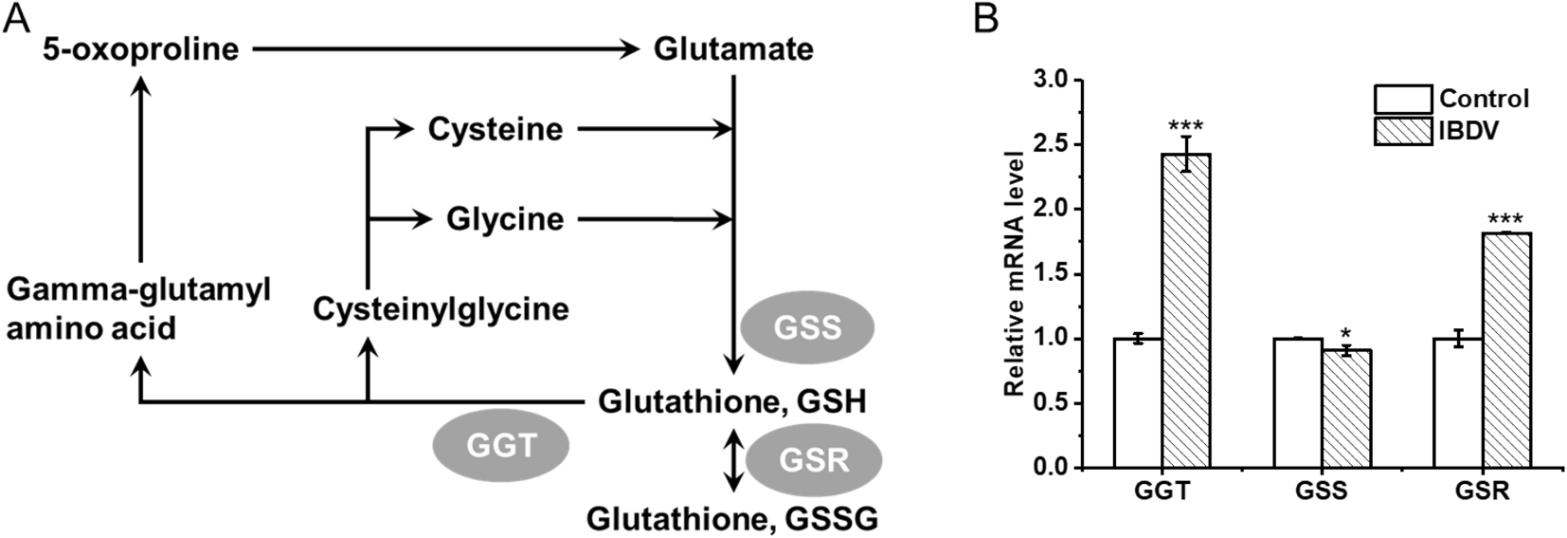
The relative mRNA levels of the key enzymes in GSH-relative pathways. **A** Glutathione metabolic pathway diagram according to metabolome. **B** The relative mRNA levels of the *ggt*, *gss*, and *gsr* genes in DF-1 cells with or without IBDV infection. *N* = 3 biological replicates and error bars represent s.d. Asterisks “*” presented the differences between the control group (without IBDV) and the experimental groups (with IBDV)

To explore the limitation of intracellular GSH concentration in DF-1 cells of IBDV infection, we examined the mRNA levels of the enzymes in related pathways, including GSH synthase (GSS) involved with GSH production, γ-glutamyltransferase (GGT) involved with GSH consumption, and glutathione reductase (GSR) which mediates the interconversion between GSH and GSSG (Fig. 3A). Compared to the control which was not infected by IBDV, the transcript levels of *ggt* and *gsr* genes were significantly increased by 2.43 ± 0.16-fold and 1.82 ± 0.08-fold, respectively, while the transcript levels of *gss* did not significantly change, indicating that the GSH utilization pathway was significantly elevated, while the GSH synthesis pathway was not significantly changed (Fig. 3B). During the antiviral oxidative stress response in mammalian cells, it is mainly GSH that plays an antioxidant role. Therefore, enhancing the synthetic pathway of GSH by overexpressing the *gss* gene could potentially improve the viral multiplication ability of DF-1 cells.

### Effect of GSS overexpression on DF-1 cell growth and IBDV propagation

**Fig. 4:**
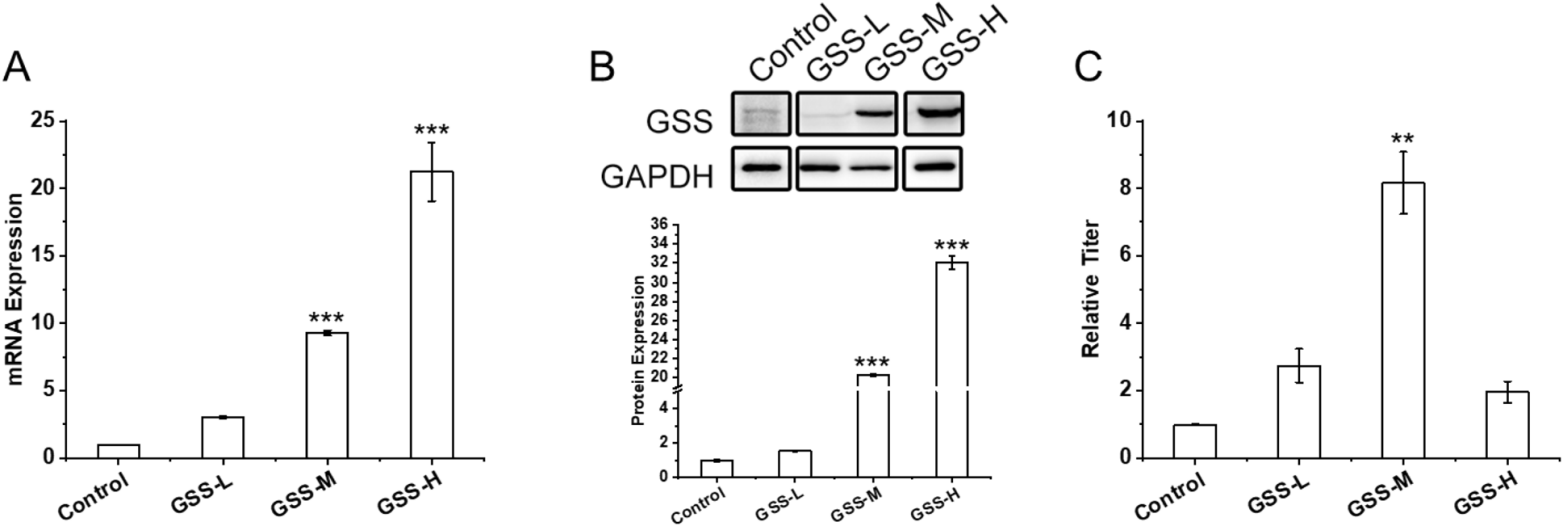
Construction of recombinant DF-1 cell lines overexpressing *gss* gene. **A** and **B** Present the *gss* transcript levels and GSS expression in the recombinant DF-1 cell lines. **C** Shows the relative IBDV titer from the recombinant cells. *N* = 3 biological replicates and error bars represent s.d. Asterisks “*” presented the differences between the control cell and the recombinant overexpressing cells. *p < 0.05, **p < 0.01, and ***p < 0.001 as determined by two-tailed *t* test

To investigate the effect of *gss* gene expression on IBDV propagation in DF-1 cells, recombinant monoclonal DF-1 cell lines overexpressing the *gss* gene were constructed and obtained by screening. According to the *gss* gene expression from low to high determined by RT-qPCR and Western Blot, three recombinant monoclonal DF-1 cell lines, GSS-L, GSS-M, and GSS-H, were selected for the subsequent study (Fig. 4A, B). The *gss* gene transcript levels of recombinant GSS-L, GSS-M, and GSS-H cell lines were increased by 3.00 ± 0.12, 9.29 ± 0.17, and 21.23 ± 2.05-fold, respectively, and the results of Western blot analysis were similar to the RT-qRCP results.

When we considered the DF-1 cells growth and IBDV propagation, the results showed the difference in the three cell lines. Compared to the control cell lines, the growth of recombinant GSS-L, GSS-M, and GSS-H cell lines were all effectively promoted, obtaining 1.50 ± 0.02, 1.18 ± 0.004, and 1.42 ± 0.06-fold higher maximum cell density and 1.33 ± 0.04, 1.76 ± 0.09, and 1.24 ± 0.01-fold higher maximum specific growth rate, respectively (Table 2; Additional file 1: Fig. S1B). And compared to control cells, the IBDV titers of recombinant GSS-L, GSS-M, and GSS-H cell lines were increased by 1.74 ± 0.50, 7.19 ± 0.93, and 0.96 ± 0.32-fold, respectively (Fig. 4C), indicating that moderate overexpression of the *gss* gene contributed to DF-1 cell growth and IBDV propagation.

**Table 2:** Growth of DF-1 cell line overexpressing *gss* gene

	Max cell density		Specific growth rate	
	10^6^cells/ml	*p*	h^−1^	*p*
Control	1.31 ± 0.01		0.021 ± 0.000	
GSS-L	1.97 ± 0.04	0.2010	0.028 ± 0.001	0.0000
GSS-M	1.55 ± 0.00	0.0000	0.037 ± 0.002	0.0030
GSS-H	1.86 ± 0.07	0.4606	0.026 ± 0.000	0.0000

The calculated mean was for triplicate measurements from two independent experiments ± s.d. and compared between the experiment group and the control group. Statistical differences were calculated using the two-tailed Student’s *t*-test in the software IBM SPSS Statistics 24

### Effect of overexpression of gss gene on redox status in DF-1 cells

**Fig. 5:**
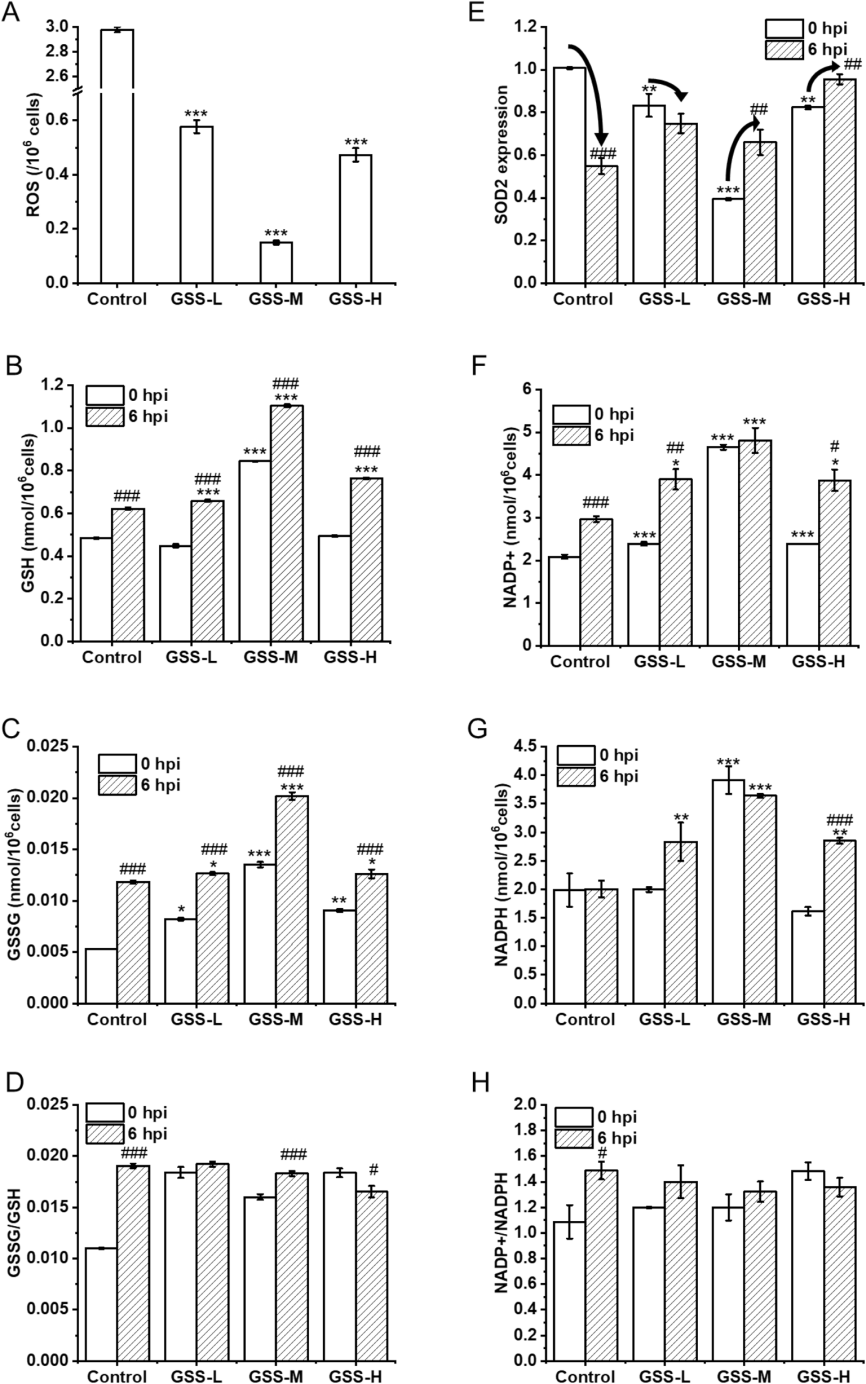
The effects of overexpression of *gss* gene on cellular redox homeostasis and anti-oxidative stress after IBDV infection in DF-1 cells. **A** Shows the fluorescence values at 488 nm of excitation wavelength and 525 nm of emission wavelength per million cells with 2,7-dichlorofluorescin diacetate treatment for the ROS detection at 0 hpi. **B** and **C** Show the intracellular concentration of GSH and GSSG in DF-1 cells at 0 hpi and 6 hpi of IBDV infection. **D** Shows the GSSG/GSH ratio calculated from **B** and **C**. **E** Shows the *sod2* transcript level in DF-1 cells at 0 hpi and 6 hpi of IBDV infection. **F** and **G** Show the intracellular concentration of NADP+ and NADPH in DF-1 cells at 0 hpi and 6 hpi of IBDV infection. **H** Shows the NADP+/NADPH ratio calculated from **F** and **G**. *N* = 3 biological replicates and error bars represent s.d. Asterisks “*” presented the differences between the control cell and the recombinant overexpressing cells. *p < 0.05, **p < 0.01, and ***p < 0.001 as determined by two-tailed t test. “#” presented the differences between 0 and 6 hpi

ROS is an important indicator to evaluate the cellular oxidative stress response. The intracellular ROS concentrations of three recombinant cell lines decreased by 80.62 ± 0.96%, 94.97 ± 0.38%, and 84.12 ± 1.25%, respectively, before being infected by IBDV (Fig. 5A). The ROS concentration of the GSS-M cell line decreased the most significantly indicating that moderate overexpression of expression of the *gss* gene was able to effectively reduce the intracellular ROS levels. Moreover, the intracellular concentrations of the related metabolites, GSH and GSSG in GSS-M cells were higher than that in control cells at 0 hpi. Therefore, the glutathione metabolism in the GSS-M cell line could be more active than that in control cell line (Fig. 5B, C). The superoxide dismutase SOD2 is required for the protection of cells from the toxicity of ROS generated during metabolism. The mRNA levels of the antioxidant gene *sod2* were decreased in all three recombinant cell lines compared to the control cell line before IBDV infection, with the most pronounced decrease in the mRNA levels of the *sod2* gene in the recombinant GSS-M cell line (Fig. 5E).

### Effect of overexpression of gss gene on cellular redox status in DF-1 cells after IBDV infection

After IBDV infection (6 hpi), the cellular redox status of DF-1 cells changed. All the cells increased the intracellular concentrations of GSH and GSSG. The GSSG/GSH (Fig. 5D) and NADP+/NADPH ratios (Fig. 4H) of control cells increased significantly, while the recombinant cell lines overexpressing the *gss* gene, the GSSG/GSH ratio and NADP+/NADPH ratio remained relatively stable. Moreover, the transcript level of the *sod2* gene decreased by 45.12 ± 4.72% in control cells, while decreased by only 8.59 ± 1.11% in GSS-L, and, respectively, increased by 26.66 ± 8.87% and 13.23 ± 3.82% in GSS-M and GSS-H at 6 hpi (Fig. 5E). The transcript levels of the antioxidant gene *sod2* in all three recombinant cell lines overexpressing the *gss* gene were higher than those in the control cell line. Therefore, overexpression of the *gss* gene strengthened both the cellular enzymatic and non-enzymatic antioxidant system when the cells were fighting against the intense oxidative stress induced by IBDV.

## Discussion

The process of viral infection can induce oxidative stress in host cells. A large number of the cellular virus infection experiments in vitro showed that severe oxidative stress occurs in host cells after infection with HIV (Thangavel et al. 2018), hepatitis C virus (Ríos-Ocampo et al. 2019), herpes simplex virus type 1 (Kristen et al. 2018), Sendai virus (Han et al. 2019), and influenza virus (Cai et al. 2003). In this study, by analyzing the metabolome changes after IBDV infection of DF-1 cells, we found a significant increase in the intensities of relevant metabolites in the glutathione metabolic pathway indicating that the IBDV infestation of DF-1 cells modulated the glutathione metabolic pathway to enhance the cellular resistance to oxidative stress in response to viral invasion. Among them, ROS is the main indicator of oxidative stress, and the production of excessive ROS overwhelms the glutathione antioxidant regulatory system and is accompanied by a significant decrease in the intracellular NADP + /NADPH ratio (Morris et al. 2013), which resulted in imbalances the redox state.

However, IBDV does not mediate the lesions in DF-1 cells once infected. DF-1 cells did not have any significant changes in cell morphology at 6 hpi, while started to develop lesions after 12 hpi (Additional file 1: Fig. S2A). Raymond Hui and Frederick Leung also found that IBDV started to replicate at 6 hpi after caIBDV infestation of DF-1 cells, and virus particle formatted at 12 hpi (Hui and Leung 2015). Moreover, we found that cellular activity began to decline significantly at 12 hpi, when the oxidative and antioxidant effects of the cells were completely imbalanced, while the intracellular redox state was still able to maintain relative homeostasis at 6 hpi (Additional file 1: Fig. S2B).

The maintenance of temporary redox homeostasis in DF-1 cells at the time of IBDV invasion facilitated the propagation of the virus at a later stage. In another study, we found that delaying IBDV-induced DF-1 cell death ultimately resulted in higher IBDV titers (Lin et al. 2020). Zhao et al*.* found that IBDV invasion in DF-1 cells induced the formation of stress granule (SG) which was an mRNA storages complex that played an important role in the innate immune response in host cells, to significantly promote IBDV replication in host cells (Zhao et al. 2020). Oxidative stress has been proven to be an inducer of SG formation, but excess ROS inhibited SG formation. Therefore, maintaining a certain concentration of intracellular GSH at the time of IBDV invasion can mitigate the damage caused by excess ROS. In addition, DF-1 cells tried to repair the imbalance of the redox state due to the intense oxidative stress response triggered by viral infection by moderately increasing GSH concentration in host cells to mitigate the onset of apoptosis and maintain the replicative environment and persistence of IBDV, this phenomenon was also found in HCV infection of Huh7.5 cells (Anticoli et al. 2019; Vasallo and Gastaminza 2015).

Endogenous overexpression of some degree of GSS likewise enhances the ability of DF-1 cells to cope with oxidative stress. In this study, we obtained three recombinant cell lines GSS-L, GSS-M, and GSS-H with low to high levels of *gss* gene overexpression by screening. We found that the intracellular ROS concentrations of all three recombinant cell lines decreased significantly compared to the control cell line before IBDV infection (0 hpi), and the recombinant GSS-M cell line showed the most significant decrease by 94.97% lower compared with the control (Fig. 4A). Therefore, the moderate overexpression of *gss* gene could effectively reduce the intracellular ROS level and improve the capacity of dealing with oxidative stress so that to make a contribution to cell growth to some extent. Although there was no significant correlation between cell growth status and *gss* gene overexpression level, the maximum cell density and maximum specific growth rate data of the two main cell growth characteristics were significantly higher in the overexpression cell lines compared to the control cell lines (Table 2).

Overexpression of the *gss* gene facilitated the transient maintenance of intracellular redox homeostasis when DF-1 cells were subjected to IBDV infestation, providing a favorable environment for viral replication. The control DF-1 cells encountered an excessive oxidative stress response and a significant imbalance in the cellular redox state, with significantly higher GSSG/GSH and NADP + /NADPH ratios at 6 hpi. The significantly elevated GSSG/GSH and NADP + /NADPH ratios of the control cell line indicated that the cells were unable to effectively regulate the excessive oxidative stress triggered by viral replication, and the oxidative and antioxidant effects were imbalanced in vivo, with the cells favoring the oxidative state. In contrast, the recombinant GSS-M cells increased the intracellular GSH and GSSG concentrations significantly (Fig. 4B, C) indicating that the cellular glutathione metabolism was enhanced and maintained the relatively stable GSSG/GSH (Fig. 4D) and NADP + /NADPH (Fig. 4H) ratio. Meanwhile, the transcript levels of the antioxidant gene *sod2* were higher in all three recombinant cell lines overexpressing the *gss* gene than in the control cell line at 6 hpi (Fig. 4E). Therefore, overexpressing *gss* enhanced both cellular non-enzymatic and enzymatic antioxidant systems against the oxidative stress induced by IBDV to maintain the redox homeostasis.

## Conclusions

In this study, by analyzing the changes in metabolome after IBDV infection of DF-1 cells, we identified the important role of glutathione metabolism on virus multiplication and suggested that the *gss* gene might be a restrictive regulator in glutathione metabolism by detecting the transcript level of the key enzymes relative to glutathione. By exogenous addition of GSH and endogenous overexpression of the *gss* gene, we have demonstrated that appropriately increasing the concentration of GSH in DF-1 cells is beneficial to improving the antioxidant stress ability of the cells, and maintaining the temporary redox homeostasis at the initial stage of IBDV infection, thus improving the viral proliferation ability in the later stage. This study provided an effective method to improve the IBDV vaccine production capacity, and sheds light on cell engineering for vaccine process that benefit from enhancing host cell resistance to oxidative stress in viral infection.

### Supplementary Information

**Additional file 1: Figure S1.** More information for the construction of recombinant DF-1 cell lines overexpressing *gss* gene. **Figure S2.** The imbalance of the redox state in DF-1 cells at 12 hpi after IBDV infection.

## Data Availability

All data sets used and analyzed are available on reasonable request.
