# Supplementary material for: Overexpression of glutathione synthetase gene improving redox homeostasis and chicken infectious bursal disease virus propagation in chicken embryo fibroblast DF-1
Source: Bioresour Bioprocess. 2023 Sep 9;10(1):60. doi: 10.1186/s40643-023-00665-0 (PMC10992565; doi:10.1186/s40643-023-00665-0)

**Additional file**

Figure S1 More information for the construction of recombinant DF-1 cell lines overexpressing *gss* gene. (A) The plasmid mapping using Snapgene. (B) The growth curves of the recombinant DF-1 cell lines overexpressing the *gss* gene.


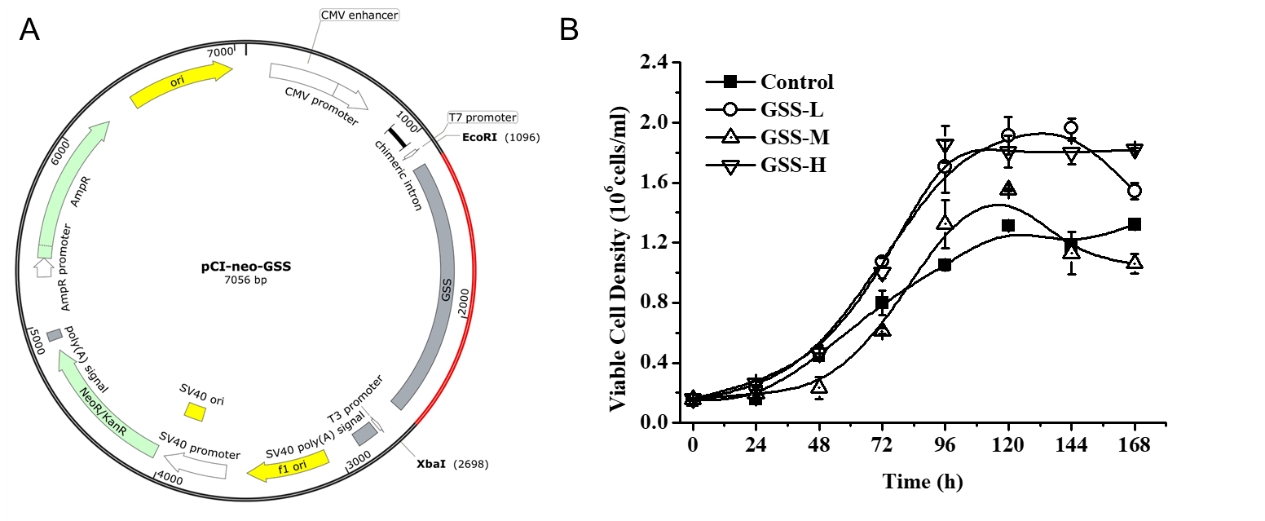


Figure S2 The imbalance of the redox state in DF-1 cells at 12hpi after IBDV infection. (A) The morphological change of DF-1 cells at 6 hpi and 12 hpi after IBDV infection. (B) The intracellular NADP+/NADPH concentrations and NADP+/NAPDH ratio in DF-1 cells after IBDV infection.


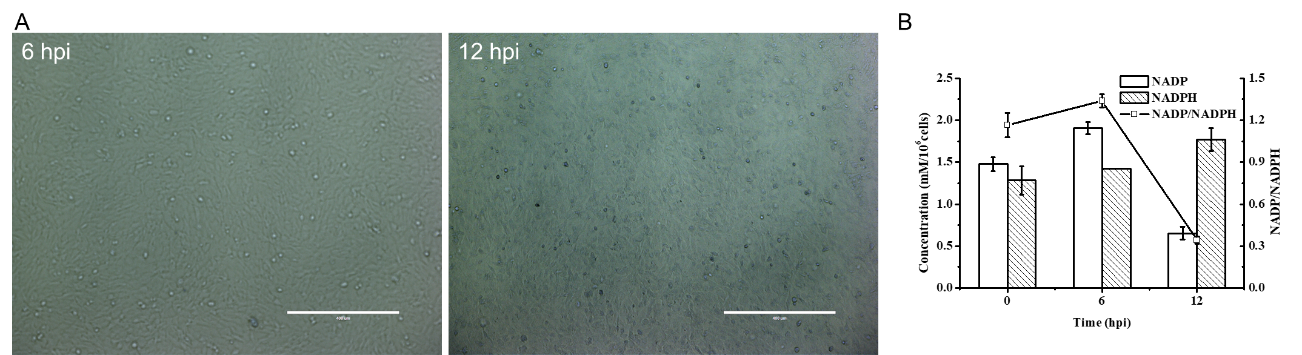

Supplement: Supplementary file 1 — Additional file 1: Figure S1. More information for the construction of recombinant DF-1 cell lines overexpressing gss gene. Figure S2. The imbalance of the redox state in DF-1 cells at 12 hpi after IBDV infection. [file 40643_2023_665_MOESM1_ESM.docx]
